# Handcycling with concurrent lower body low-frequency electromyostimulation significantly increases acute oxygen uptake: implications for rehabilitation and prevention

**DOI:** 10.7717/peerj.13333

**Published:** 2022-05-18

**Authors:** Ludwig Rappelt, Steffen Held, Lars Donath

**Affiliations:** Department of Intervention Research in Exercise Training, German Sport University Cologne, Cologne, Germany

**Keywords:** Electrical muscle stimulation, EMS, Cardiorespiratory fitness, Endurance, SCI, Spinal cord injury, Wheelchair

## Abstract

**Background:**

Acute increases in exercise-induced oxygen uptake (V̇O_2_) is crucial for aerobic training adaptations and depends on how much muscle mass is involved during exercising. Thus, handcycling is per se limited for higher maximal oxygen uptakes (V̇O_2_max) due to restricted muscle involvement. Handcycling with additional and simultaneous application of low-frequency electromyostimulation (EMS) to the lower extremities might be a promising stimulus to improve aerobic capacity in disabled and rehabilitative populations.

**Method:**

Twenty-six healthy young adults (13 female, age: 23.4 ± 4.5 years, height: 1.77 ± 0.09 m, mass: 71.7 ± 16.7 kg) completed 4 ×10 minutes of sitting (SIT), sitting with concurrent EMS (EMS_SIT), handcycling (60 rpm, 1/2 bodyweight as resistance in watts) (HANDCYCLE) and handcycling with concurrent EMS of the lower extremities (EMS_HANDCYCLE). During EMS_SIT and EMS_HANDCYCLE, low frequency EMS (impulse frequency: 4Hz, impulse width: 350 µs, continuous stimulation) was applied to gluteal, quadriceps and calf muscles. The stimulation intensity was selected so that the perceived pain could be sustained for a duration of 10 minutes (gluteus: 80.0 ± 22.7 mA, quadriceps: 94.5 ± 20.5 mA, calves: 77.5 ± 19.1 mA).

**Results:**

Significant mode-dependent changes of V̇O_2_ were found (*p* < 0.001, *η*_p_^2^ = 0.852). Subsequent post-hoc testing indicated significant difference between SIT *vs.* EMS_SIT (4.70 ± 0.75 *vs.* 10.61 ± 4.28 ml min^−1^ kg^−1^, *p* < 0.001), EMS_SIT *vs.* HANDCYCLE (10.61 ± 4.28 *vs.* 13.52 ± 1.40 ml min^−1^ kg^−1^, *p* = 0.005), and between HANDCYCLE *vs.* EMS_HANDCYCLE (13.52 ± 1.40 *vs.* 18.98 ± 4.89 ml min^−1^ kg^−1^, *p* = 0.001).

**Conclusion:**

Handcycling with simultaneous lower body low-frequency EMS application elicits notably higher oxygen uptake during rest and moderately loaded handcycling and may serve as an additional cardiocirculatory training stimuli for improvements in aerobic capacity in wheelchair and rehabilitation settings.

## Introduction

The highest amount of oxygen that can be utilized during severe exercise is defined as maximum oxygen uptake (V̇O_2_max) (Bassett & Howley, 2000). Along with time trial performance and exercise economy (Paavolainen, Nummela & Rusko, 2000), V̇O_2_max is considered a meaningful physiological variable to evaluate cardiorespiratory and -circulatory capacity (Bassett & Howley, 2000). Acute oxygen uptake (V̇O_2_) mainly depends on how much muscle mass is involved in a certain exercise (Poole & Richardson, 1997). Consequently, V̇O_2_ uptake is per se diminished during arm exercises (*e.g.*, handcycling) compared with leg exercises (*e.g.*, conventional cycle ergometer) (Astrand, Guharay & Wahren, 1968; Franklin et al., 1983). As a result, arm exercises might be less effective than leg exercises for developing and maintaining high levels of oxygen uptake (Franklin, 1989). This is a particular challenge for people with spinal cord injuries (SCI) trying to increase their V̇O_2_max for health (Kaminsky et al., 2013) or sport-specific performance purposes (Bassett & Howley, 2000).

Electromyostimulation (EMS) might be employed as a complementary training method suited for people with SCI or lower body injuries and athletes relying on a wheelchair: EMS applied to lower extremity muscles has been shown to prevent muscle atrophy in SCI patients (Baldi et al., 1998), induce notable strength and endurance adaptations (Sloan et al., 1994), and enhance metabolic or hemodynamic responses (Hooker et al., 1992; Hooker et al., 1995; Phillips & Burkett, 1995). Acute responses following EMS application to the lower extremity muscles in wheelchair depending populations include increased oxygen uptake and cardiac output (Hooker et al., 1992; Phillips & Burkett, 1995; Mutton et al., 1997; Paulson et al., 2014). These studies were, however, often conducted with small sample sizes, lacking randomization and adequate control conditions. Additionally, in these studies the different muscle groups of the legs were often stimulated in a specific computer-controlled pattern to initiate leg movement (functional electrical stimulation; FES), requiring therefore special equipment. In a recent study (Bakkum et al., 2015), 20 SCI patients were randomly assigned to either handcycling or handcycling with additional FES of the legs at a maximum tolerable intensity (0–150 mA). Both groups exercised twice weekly for 18–32 min at an intensity of 65–75% of heart rate reserve over a period of 16 weeks. However, no significant improvements in peak oxygen uptake (V̇O_2_peak) were found in either group in a wheelchair ramp test. It was hypothesized, that the low training frequency and volume lead to the lack of improvements (Bakkum et al., 2015). Further, a systematic review regarding the metabolic efficiency of FES-induced cycling movements revealed a low efficiency for this stimulation method (Hunt et al., 2012). Therefore, alternative stimulation patterns compared to FES were recommended (Hunt et al., 2012).

In terms of stimulation frequencies, electrical stimulation of the lower extremities was often applied at higher frequencies (*e.g.*, 30–35 Hz, (Hooker et al., 1992; Phillips & Burkett, 1995; Mutton et al., 1997)). However, a more recent study indicated that low-frequency stimulation induces lower fatigue and increases the time to exhaustion (Downey et al., 2015), which may allow for a longer training duration and therefore a higher volume. Further, Petrie and colleagues (Petrie, Suneja & Shields, 2015) found a higher increase in key metabolic transcript factors after a single session of low-frequency stimulation at 5 Hz compared to pulse-matched stimulation at 20 Hz. Therefore, potentially higher training adaptation potential occurs at a lower stimulation frequency.

Against this background, we aimed to elucidate, whether an acute moderate handcycle training load can be significantly increased by a concurrent application of low-frequency EMS with a continuous stimulation pattern on the muscles of the lower extremities. We hypothesized that the concurrent application of EMS on the lower limb muscles will lead to significantly and meaningful higher exercise induced oxygen uptake. These findings may have relevant impact on handcycling training designs that are intended to improving endurance performance of wheelchair or rehabilitation population.

## Materials & Methods

### Participants

A previously conducted power analysis (*α* = 0.05, study power (1-*β*-error) = 0.80, *r* = 0.5, effect size *η*_p_^2^ = 0.06 (*f* = 0.25)), with G*Power (Version 3.1.9.7, University Kiel, Germany), revealed a required sample size of *n* = 23 participants. Assuming low dropouts, 26 healthy young adults (13 female, age: 23.4 ± 4.5 years, height: 1.77 ± 0.09 m, body mass: 71.7 ± 16.7 kg) were enrolled in this study. Inclusion criteria were as follows: (I) aged between 18 and 40 years, (II) no cardiovascular diseases (World Health Organization, 2021), (III) no metabolic diseases (*e.g.*, diabetes mellitus), (IV) no acute or chronic skin diseases or injuries. Participants were instructed to avoid strenuous exercise 72 h prior to their study attendance. The study met the requirements of the declaration of Helsinki and was approved by the ethical committee of the German Sport University Cologne (163/2020). All participants signed an informed written consent prior to the start of the study.

### Study design

The acute intervention consisted of a single lab visit. After assessing anthropometric data, participants were shortly familiarized with EMS. For familiarization, EMS stimulation at the individual maximal tolerable pain threshold (see Data acquisition) was applied for a period of 2–3 min. Thereafter, participants completed the acute intervention protocol consisting of four 10-min intervals interspersed with 5-min of passive rest. During these 4 × 10-min sequences, all participants completed a bout of sitting (SIT), sitting with concurrent EMS of the legs (EMS_SIT), handcycling (HANDCYCLE) and handcycling with concurrent EMS of the legs (EMS_HANDCYCLE). The acute intervention started for all participants with a 3-min rest phase, followed by the first two conditions (SIT & EMS_SIT). The conditions of HANDCYCLE and EMS_HANDCYCLE were subsequently completed in a randomized order.

### Data acquisition

During EMS_SIT and EMS_HANDCYCLE low frequency EMS (impulse frequency: 4 Hz, impulse width: 350 µs, continuous stimulation) was applied to the lower extremities. A total of 6 surface electrodes (MIHA BODYTEC II; miha bodytec GmbH, Gersthofen, Germany) were wrapped around the buttocks, thighs, and calves of the participants. Depending on the body dimensions, different sizes of belt electrodes (small/medium/large) were applied to provide stimulation of the glutei (length × height: 13 cm × 10 cm), the quadriceps/hamstring muscles (35.5–60.5 cm × 4 cm) as well as the triceps surae (20.5–32.5 cm × 4 cm). During the initial familiarization phase, participants were instructed to reach a stimulation intensity, at which the perceived pain could be tolerated over a duration of 10 min (individual maximal tolerable pain threshold; IPT). In order to determine the IPT, the main controller at the EMS device (MIHA BODYTEC II; miha bodytec GmbH, Gersthofen, Germany) was regulated to its maximal intensity (100%, 120 mA). Subsequently, starting at the buttocks and continuing with the thighs and calves, IPT was determined for each pair of electrodes separately, using the respective individual controller at the EMS device (buttocks: 80.0 ± 22.7 mA, thighs: 94.5 ± 20.5 mA, calves: 77.5 ± 19.1 mA). The intensity that reached the muscles cannot be precisely determined due to differences in the resistance of tissue structures (Lake, 1992).

Load during HANDCYCLE and EMS_HANDCYCLE consisted of handcycling performed on a modified cycle ergometer (SRM Ergo; SRM Training Systems GmbH, Germany). This ergometer was modified for arm crank exercise by replacing its pedals with handles and raising its frame so that the handles could be rotated in a sitting position. The cranks were mounted in anti-phase to the ergometer and had a length of 17.5 cm. Further, the seat height and position were individually adjusted for each participant to ensure a standardized position of the crank axis relative to the shoulder. The load during handcycling was set at 50% of the respective participants body mass (35.9 ± 8.4 W) and the participants were instructed to maintain a target cranking cadence of 60rpm with visual feedback provided by the corresponding software of the ergometer.

Throughout the whole session breath-by-breath gas exchange data were continuously collected using a validated spirometric system (Zan Oxi 600; Zan Messgeräte, Oberthulba, Germany). The spirometric system was calibrated prior to each test, following the manufacturer’s recommendations. Additionally, heart rate (HR) was recorded using a chest strap (H7; Polar Electro, Kempele, Finnland). Total ventilation volume (VE), carbon dioxide output (V̇CO_2_), V̇O_2_ and HR were averaged over the last 3 min for each of the four conditions. Further, the respiratory exchange ratio (RER) was calculated by dividing V̇CO_2_ by V̇O_2_.

Prior, and immediately after each of the four conditions, 20 µl of capillary blood were collected from the earlobe to determine the blood lactate level (Biosen C-Line; EKF Diagnostic Sales, Magdeburg, Germany). Subsequently, the difference between the pre- and post-value for each condition was computed (ΔLactate). Due to technical problems, lactate samples of two participants could not be analyzed.

### Statistical analysis

All data are provided as mean value ± standard deviation (SD). Parameters were initially visually inspected for normal distribution and variance homogeneity. To examine the “mode” differences (SIT *vs.* EMS_SIT *vs.* HANDCYCLE *vs.* EMS_HANDCYCLE) repeated measures analyses of variance (rANOVA) were separately conducted for the respective outcome measures (V̇O_2_, V̇CO_2_, VE, RER, ΔLactate and HR). The effect sizes for the rANOVA are provided as partial eta squared (*η*_p_^2^) with ≥0.01, ≥0.06, ≥0.14 indicating small, moderate, and large effects, respectively (Cohen, 1988). Further, standardized mean differences (SMDs) for pairwise effect size comparison were calculated as the differences between the means divided by the pooled standard deviations (trivial: — SMD —< 0.2, small: 0.2 ≤ — SMD —< 0.5, moderate: 0.5 ≤— SMD —< 0.8, large: — SMD —≥ 0.8) (Cohen, 1988). All statistical analyses were performed using R in its integrated development environment RStudio (Version 1.2.5033, R version 3.6.2).

## Results

**Figure 1:**
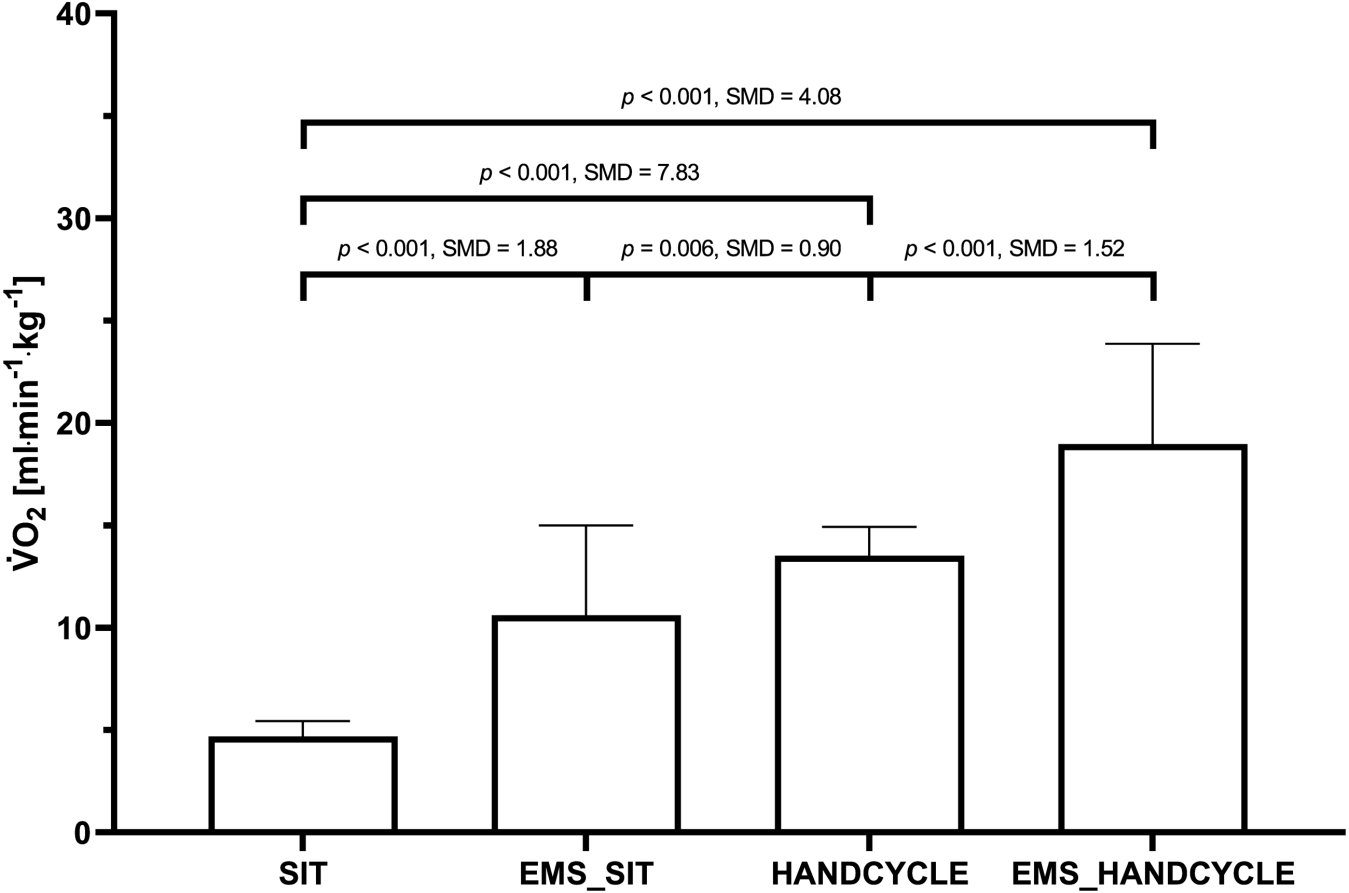
Comparison of oxygen uptake during the different conditions. Mean value ± standard deviation of oxygen uptake (V̇O_2_max) during sitting (SIT), sitting with concurrent electrostimulation of the legs (EMS_SIT), handcycling (HANDCYCLE) and handcycling with concurrent electrostimulation of the legs (EMS_HANDCYCLE). *p*-values and standardized mean differences (SMD) of pairwise comparisons are indicated.

The rANOVA showed large and statistically significant “mode” differences for V̇O_2_ (F(3, 75) = 143.35, *p* < 0.001, ${\eta }_{\mathrm{p}}^{2}=0.852$). Post-hoc testing indicated a statistically significant difference between SIT and EMS_SIT (4.70 ± 0.75 *vs.* 10.61 ± 4.28 ml min^−1^ kg^−1^, *p* < 0.001), EMS_SIT and HANDCYCLE (10.61 ± 4.28 *vs.* 13.52 ± 1.40 ml min^−1^ kg^−1^, *p* = 0.005), as well as between HANDCYCLE and EMS_HANDCYCLE (13.52 ± 1.40 *vs.* 18.98 ± 4.89 ml min^−1^ kg^−1^, *p* = 0.001) (Fig. 1).

**Figure 2:**
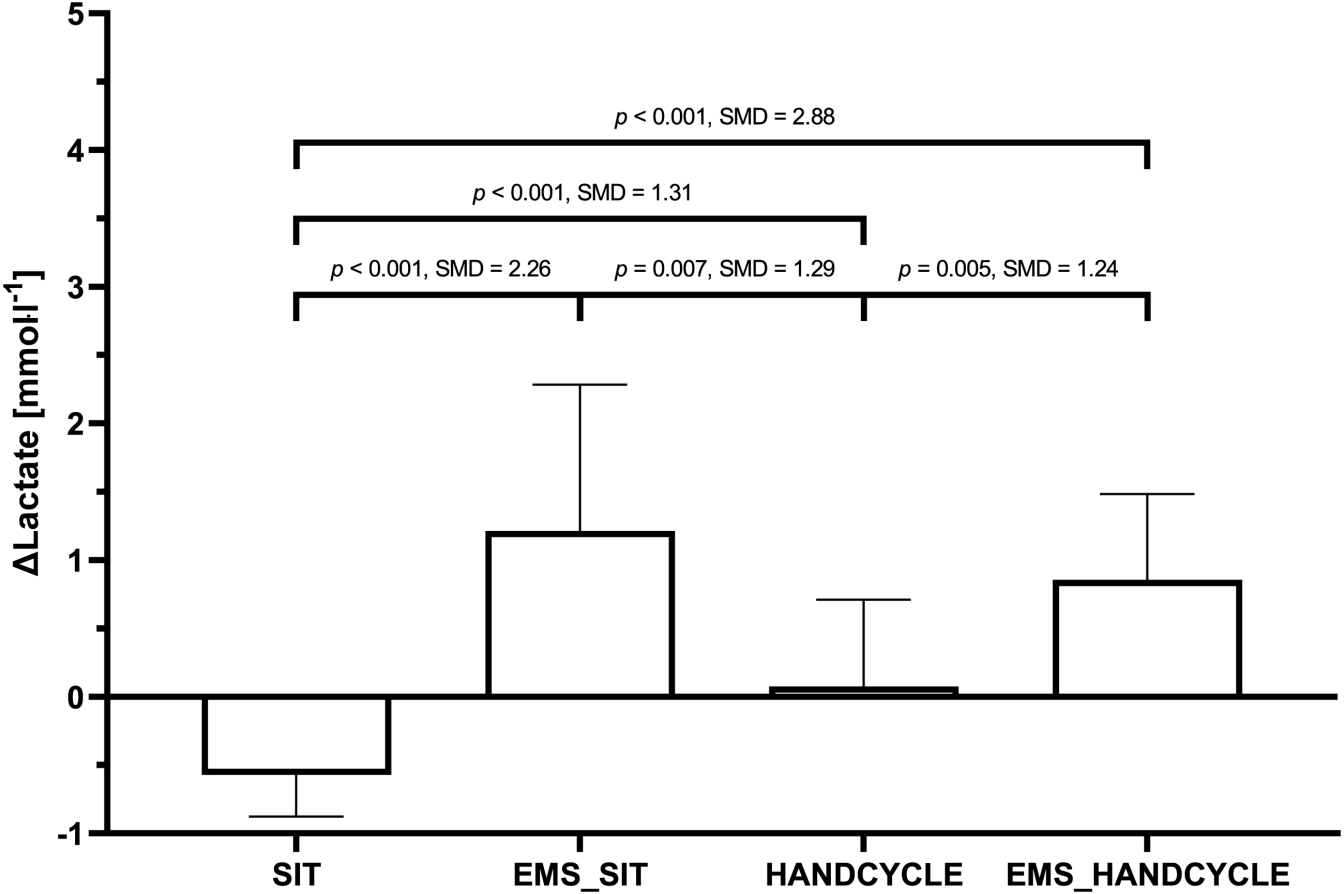
Comparison of changes in blood lactate concentration between the different conditions. Mean value ± standard deviation of POST-PRE differences in blood lactate Level (Δ Lactate) during sitting (SIT), sitting with concurrent electrostimulation of the legs (EMS_SIT), handcycling (HANDCYCLE) and handcycling with concurrent electrostimulation of the legs (EMS_HANDCYCLE). *p*-values and standardized mean differences (SMD) of pairwise comparisons are indicated.

Further, large and statistically significant “mode” differences were also found for ΔLactate (F(3, 69) = 26.54, *p* < 0.001, ${\eta }_{\mathrm{p}}^{2}=0.536$). Post-hoc testing did show statistically significant difference between SIT and EMS_SIT (−0.57 ± 0.31 *vs.* 1.21 ±1.07 mmol l^−1^, *p* < 0.001), EMS_SIT and HANDCYCLE (1.21 ± 1.07 *vs.* 0.08 ± 0.63 mmol l^−1^, *p* = 0.007), as well as between HANDCYCLE and EMS_HANDCYCLE (0.08 ± 0.63 *vs.* 0.86 ± 0.63 mmol l^−1^, *p* = 0.005) (Fig. 2).

The rANOVA did also reveal large and significant “mode” differences for V̇CO_2_ (F(3, 75) = 97.52, *p* < 0.001, ${\eta }_{\mathrm{p}}^{2}=0.796$), VE (F(3, 75) = 29.75, *p* < 0.001, ${\eta }_{\mathrm{p}}^{2}=0.543$), RER (F(3, 75) = 98.57, *p* < 0.001, ${\eta }_{\mathrm{p}}^{2}=0.798$), and HR (F(3, 75) = 160.84, *p* < 0.001, ${\eta }_{\mathrm{p}}^{2}=0.865$) (Table 1).

**Table 1:** Comparison of performance parameters. Comparison between sitting (SIT), sitting with concurrent electromyostimulation of the legs (EMS_SIT), handcycling (HANDCYCLE), and handcycling with concurrent electromyostimulation of the legs (EMS_HANDCYCLE).

**Parameter**	**SIT**	**EMS_SIT**	**HANDCYCLE**	**EMS_HANDCYCLE**
V̇O_2_ [l min^−1^]	0.27 ± 0.08^+^	0.70 ± 0.38	0.85 ± 0.20	1.23 ± 0.44^+^
VE [l min^−1^]	8.94 ± 2.20^+^	20.08 ± 9.85^**^	25.11 ± 5.70	35.75 ± 13.31^+^
RER [AU]	0.80 ± 0.06^+^	0.89 ± 0.07	0.87 ± 0.05	0.89 ± 0.04^*^
HR [min^−1^]	70.0 ± 9.7^+^	84.7 ± 17.0^***^	103.0 ± 14.7	114.8 ± 18.5^+^

**Notes.**

+ Significantly different from any other condition (*p* < 0.001).

* Significantly different from HANDCYCLE (*p* < 0.05).

** Significantly different from HANDCYCLE (*p* < 0.01).

*** Significantly different from HANDCYCLE (*p* < 0.001).

## Discussion

This controlled crossover-study aimed at elucidating whether the combination of handcycling and complementary EMS of the lower extremities induces higher oxygen uptake than either handcycling or EMS of the lower extremities alone. Our main findings indicate that EMS applied to the lower extremities leads to a meaningful acute increase of oxygen demand in both sitting and submaximal handcycling compared to sitting and submaximal handcycling without EMS. During both sitting and handcycling with EMS a significant onset of blood lactate level was observed, while during sitting and handcycling without EMS blood lactate levels were found to decline or maintain their level, respectively.

The increases in oxygen demand found in this study between sitting and sitting with EMS are slightly lower, but in line with the results reported by Banerjee and colleagues (Banerjee et al., 2005) (+0.4 ± 0.3 *vs.* +0.7 ± 0.2 l min^−1^). In their study, cardiopulmonary gas exchange was recorded from 10 healthy participants lying supine with their buttocks and thighs stimulated by EMS with increasing intensity (4 × 3 min with 30–120 mA). Although the total area of the electrodes was smaller in the study by Banerjee and colleagues (Banerjee et al., 2005) than in the present study (between −15% and −40%) and only the buttocks and thighs of the participants were stimulated, higher rates of oxygen consumption were reported at maximum stimulation intensity (120 mA). These differences might be attributed to the lower stimulation intensity used in our study (buttocks: 80.0 ± 22.7 mA, thighs: 94.5 ± 20.5 mA, calves: 77.5 ± 19.1 mA), as Banerjee and colleagues also reported that oxygen consumption increases with increasing stimulation intensity (Banerjee et al., 2005). Therefore, it seems plausible to assume, that at least at rest, the level of stimulation intensity affects oxygen consumption.

Maximal oxygen uptake on handcycle ergometers is reduced by approximately 20–36% compared to conventional (leg-operated) bicycle ergometers (Astrand, Guharay & Wahren, 1968; Fardy, Webb & Hellerstein, 1977; Franklin et al., 1983). In order to maintain or develop cardiovascular fitness, a sufficient stimulus must be imposed (Pollock et al., 1998). In this context, arm exercises are considered less efficient and effective than leg exercises (Franklin, 1989). In the present study, the concurrent application of EMS on the legs acutely increased the oxygen demand by 39.7 ± 30.0% (0.97 ± 0.21 l min^−1^ to 1.36 ± 0.44 l min^−1^) compared to handcycling without additional EMS stimulation. In SCI patients, working at maximum intensity on an arm-crank ergometer, increases in V̇O_2_ demand of 35% were found when additional leg cycle ergometry was electrically induced (Figoni et al., 1989). However, this stimulation differed from the present study as movement of the legs was initiated by computer-controlled electromyostimulation of the paralyzed leg muscles, whereas the legs were at rest in the present study. Nevertheless, a different study found a significant increase of oxygen demand from 0.75 ±0.11 to 0.83 ± 0.10 l min^−1^ at submaximal working intensity (hand cranking at 50% of V̇O_2_max) by applying concurrent electrical stimulation of the paralyzed leg muscles of six participants with SCI without initiating movement (Thomas, Davis & Gass, 1992). Therefore, although the stimulation of the lower extremities was performed differently across these studies, the reported results are in line with our findings. It thus seems that during handcycling a concurrent application of EMS on the legs increases the oxygen demand regardless of the working intensity.

Regarding long-term adaptations, the underlying mechanisms for an increased aerobic capacity after the application of low-frequency EMS remain unclear (Deley & Babault, 2014). Changes in the mitochondrial content, myosin heavy chain isoforms and increased peripheral perfusion have been suggested as possible underlying mechanisms (Nuhr et al., 2003; Dobsák et al., 2006). In able-bodied individuals, the influence of EMS on cardiovascular adaptations has been reported several times. Miyamoto and colleagues found statistically significant improvements in V̇O_2_max after 4 weeks of EMS stimulation (30 min, 4 times per week, Impulse frequency: 4 Hz, Impulse width: 250 µs, continuous stimulation) of the lower extremities during rest (Miyamoto et al., 2016). Similarly, Nuhr and colleagues (2003) confirmed in a 10-week RCT (4 h EMS per day, 7 times per week, 15 Hz, 500 µs impulse width) that low-frequency EMS improves maximal aerobic oxidative capacity and V̇O_2_ at the anaerobic thresholds. Conversely, continuous cycling of 60 min at 60% peak power output (PPO)) with concurrent EMS applied to the lower extremities (Impulse frequency: 80 Hz, Impulse width: 400 µs, Impulse-Stimulation-to-Rest-Ratio: 10s:2s) did not result in superior improvements of endurance performance compared to continuous cycling alone after 4 weeks of intervention in healthy, moderately trained young males (Mathes et al., 2017). From an interventional training perspective, it seems that, at least in healthy subjects, the application of continuous electric stimulation at very low frequency may be superior in its adaptational potential. In SCI patients, lower extremity EMS with simultaneous arm activity (handcycle ergometer) was found to significantly increase V̇O_2_max (Mutton et al., 1997). However, this study was designed as a non-randomized control trial with the enrolled participants acting as their own control. Therefore, further randomized control trials are warranted to elucidate to which extent the simultaneous application of EMS to the lower extremities during handcycling leads to an increase in cardiopulmonary capacity in SCI patients.

The observed lactate kinetics during sitting and handcycling with EMS could be explained by the following aspects: EMS is considered to recruit motor units in a nonselective pattern, innervating both slow-twitch and fast-twitch muscle fibres (Gregory & Bickel, 2005). The fibre type distribution in the human muscle seems to be non-random, with slow-twitch fibres being predominantly allocated in deeper regions of the muscle and fast-twitch fibres at the surface (Lexell, Henriksson-Larsén & Sjöström, 1983). Therefore, fast-twitch fibres are in near proximity to the EMS surface electrodes and in turn more likely to be stimulated. As fast-twitch muscle fibers are known to produce lactate at a higher rate than slow-twitch muscle fibers (Tesch & Karlsson, 1977), this leads to a higher blood lactate level. The higher metabolic load induced by the elevated intramuscular lactate concentration may influence long-term mitochondrial adaptations (Takahashi et al., 2019). Depending on training intensity, lactate produced in one limb can be transported and metabolized in others (Van Hall et al., 2003). As lactate can be reused during aerobic exercise (Brooks, 2018), handcycling at a low intensity may have allowed lactate produced in the legs to be metabolized in the working muscles of the arms. This may explain the slightly lower increase in lactate during handcycling with EMS compared to sitting with EMS. However, this remains speculative, as the blood lactate level was determined only at the earlobe and therefore no conclusion can be drawn about the net release and net uptake of selective muscle groups.

A limitation of this study that needs to be addressed is that the muscle mass of the legs in able-bodied persons is most likely significantly larger than in SCI patients. Also, the proportion of muscle fibre types seem to differ between paralyzed and non-paralyzed muscles with paralyzed muscles showing a higher proportion of type IIx fibres (Malisoux et al., 2007). Therefore, the cardiovascular and -respiratory response to the application of EMS could be less prominent in this population. However, the functional performance of paralyzed muscle fibres does not seem to differ from muscle fibres with normal neuromuscular activity (Malisoux et al., 2007). Also, in terms of adaptational processes, electrical stimulation has been shown to upregulate metabolically active genes in paralyzed muscles and non-paralyzed muscles to a similar degree (Gorgey et al., 2017). Further, considering the dose–response relationship and the ability to sustain higher stimulation intensities of SCI patients, the lower stimulated muscle mass could be compensated by a higher EMS intensity. Therefore, the results found in healthy participants can also be applied to SCI patients.

## Conclusions

In conclusion, the application of low-frequency EMS of the lower extremities acutely leads to a significant and meaningful increase in oxygen uptake during rested (sitting) and loaded (handcycling) conditions. Concurrent application of EMS during handcycling may help increasing the hemodynamic response leading therefore to greater adaptations in V̇O_2_max. This is of particular interest for persons with spinal cord injuries. Future research should evaluate the long-term adaptations in V̇O_2_max induced by the simultaneous application of low-frequency EMS to the lower extremities during handcycling in adequately powered randomized control trials in persons with spinal cord injuries on individual levels.

## Supplemental Information

10.7717/peerj.13333/supp-1 Supplemental Information 1 Raw data Raw data for each participant (load, heart rate, spirometric data) and R-Scripts for data processing (as described in the material & method section under “Data aquisition”), and EMS intensity and blood lactate concentration.

## Additional Information and Declarations

### Data Availability

The following information was supplied regarding data availability: The raw data is available in the Supplementary File.
